# Author Correction: The prognostic effects of somatic mutations in ER-positive breast cancer

**DOI:** 10.1038/s41467-018-07407-3

**Published:** 2018-11-14

**Authors:** Obi L. Griffith, Nicholas C. Spies, Meenakshi Anurag, Malachi Griffith, Jingqin Luo, Dongsheng Tu, Belinda Yeo, Jason Kunisaki, Christopher A Miller, Kilannin Krysiak, Jasreet Hundal, Benjamin J Ainscough, Zachary L. Skidmore, Katie Campbell, Runjun Kumar, Catrina Fronick, Lisa Cook, Jacqueline E. Snider, Sherri Davies, Shyam M. Kavuri, Eric C. Chang, Vincent Magrini, David E. Larson, Robert S Fulton, Shuzhen Liu, Samuel Leung, David Voduc, Ron Bose, Mitch Dowsett, Richard K. Wilson, Torsten O. Nielsen, Elaine R Mardis, Matthew J. Ellis

**Affiliations:** 1 McDonnell Genome Institute, Washington University School of Medicine, St. Louis 63108, MO USA; 2 Department of Medicine, Division of Oncology, Washington University School of Medicine, St. Louis 63110, MO USA; 3 Siteman Cancer Center, Washington University School of Medicine, St. Louis 63110, MO USA; 4 Department of Genetics, Washington University School of Medicine, St. Louis 63110, MO USA; 5 Lester and Sue Smith Breast Center and Dan L. Duncan Cancer Center, Baylor College of Medicine, Houston 77030, TX USA; 6 Departments of Medicine and Molecular and Cellular Biology, Baylor College of Medicine, Houston 77030, TX USA; 7 Division of Biostatistics, Washington University School of Medicine, St. Louis 63110, MO USA; 8 Genetic Pathology Evaluation Centre, University of British Columbia, Vancouver V6H 3Z6, Canada; 9 Institute of Cancer Research, London SM2 5NG, UK; 10 Present Address: Nationwide Children’s Hospital and Department of Pediatrics, The Ohio State University College of Medicine, Columbus 43205, OH USA

Correction to: *Nature Communications*; 10.1038/s41467-018-05914-x; published online 04 September 2018

The original version of this Article contained errors in the depiction of confidence intervals in the NF1 BCSS data illustrated in Fig. 3b. These have now been corrected in both the PDF and HTML versions of the Article. The incorrect version of Fig. 3b is presented below.

**Figure 1:**
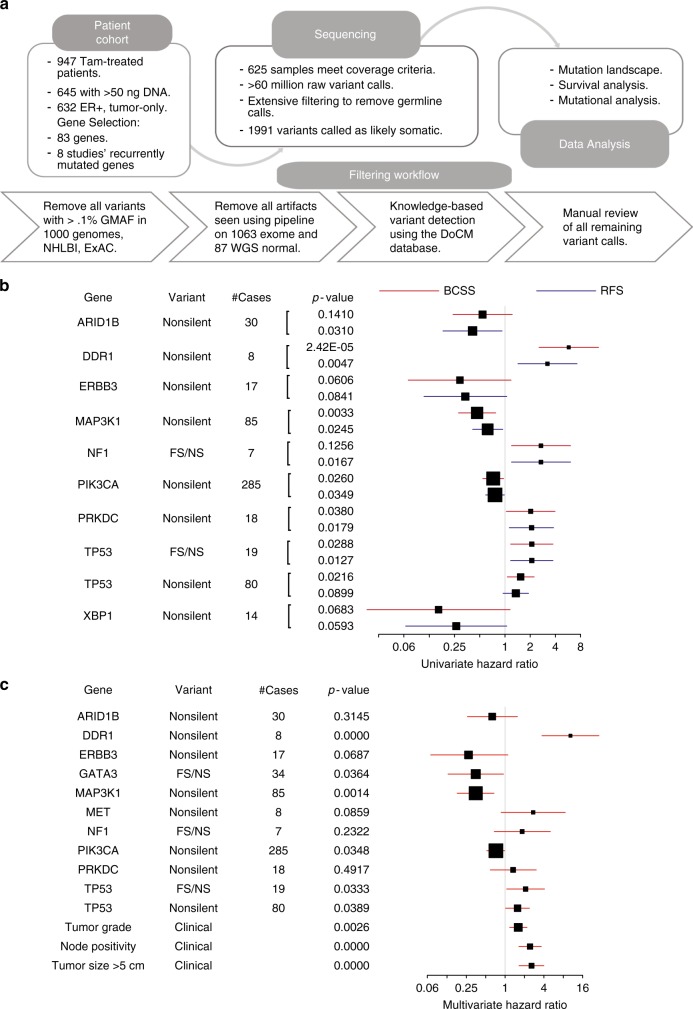
Fig. 3

