# Supplementary material for: The prognostic effects of somatic mutations in ER-positive breast cancer
Source: Nat Commun. 2018 Sep 4;9:3476. doi: 10.1038/s41467-018-05914-x (PMC6123466; doi:10.1038/s41467-018-05914-x)
Supplement: Supplementary file 1 — Supplementary Information [file 41467_2018_5914_MOESM1_ESM.pdf]

Supplementary Figures

Supplementary Figure 1. Percent bases covered by coverage cutoff

The figure below summarizes the percent of the bases (x-axis) that are covered for each sample at several depth cutoffs (y-axis) from 1X to 40X for TAM, POLAR and MA12 cohorts. For example, there were 7 of 632 samples that were deemed to have failed sequencing because they did not reach the minimum requirement of 80% covered at greater than 20X. This represents a 98.8% success rate. Most samples far exceeded this minimum cutoff in all three cohorts.

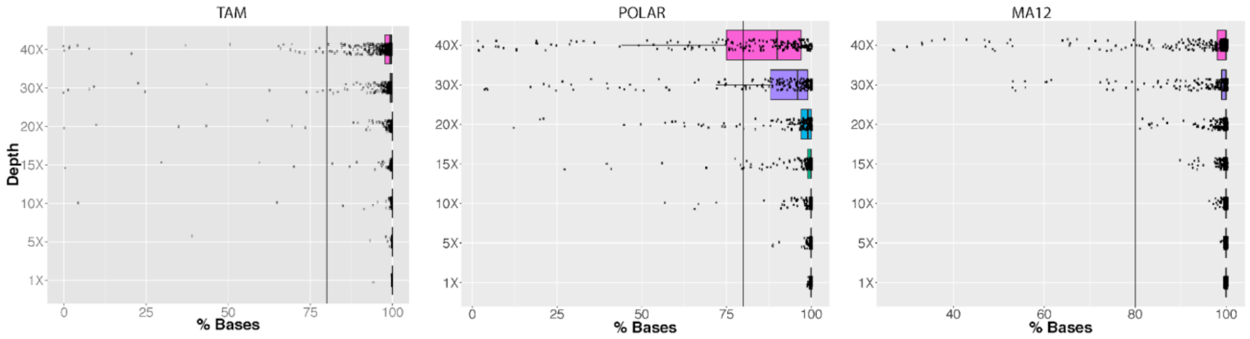

Supplementary Figure 2. Percent alignment by category

This plot summarizes the percentage of the reads that fell into each alignment category as unique or duplicate alignments, on- or off-target for the exons of the 83-gene panel, or unaligned for the TAM, POLAR and MA12 cohorts. The box borders show first quartile, mean, and third quartile.

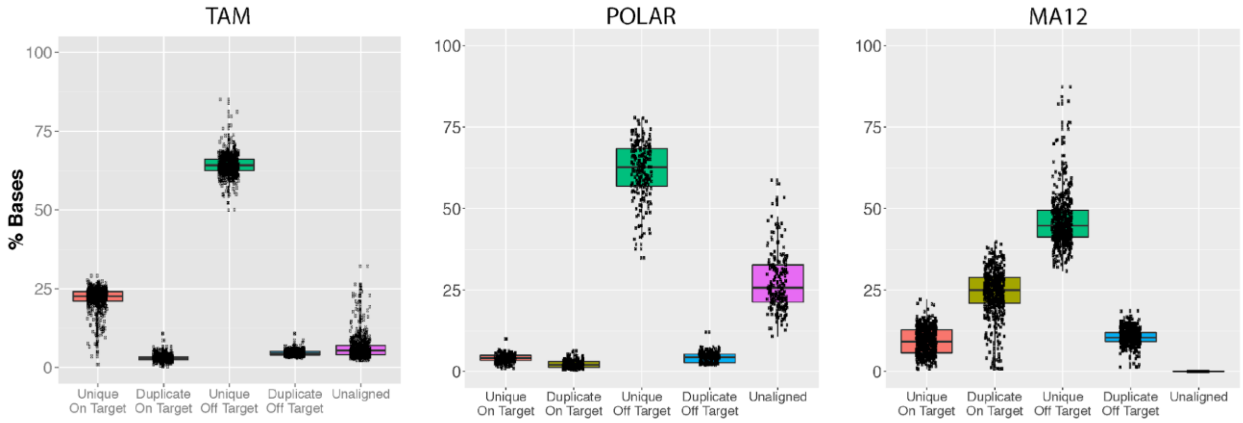

Supplementary Figure 3. Mean depth and percent duplicates by library input and time since diagnosis

The mean read depth (panels A and C) and on-target duplication rates (panels B and D) are plotted against library input DNA (ng) (panels A and B) and time since diagnosis (years) (panels C and D) respectively for the TAM cohort. Mean depth was positively correlated with input DNA and negatively correlated with time since diagnosis (approximate age of sample). Conversely, duplication rates were negatively correlated with input DNA and positively correlated with sample age. Where input DNA amounts allowed 8 instead of 10 PCR cycles (minimum 150ng required) mean depths were generally higher and duplication rates lower. However, despite these trends, overall metrics were excellent with an average of 135.8X coverage and 3.0% duplicate rate despite the generally low input amounts and old age of samples.

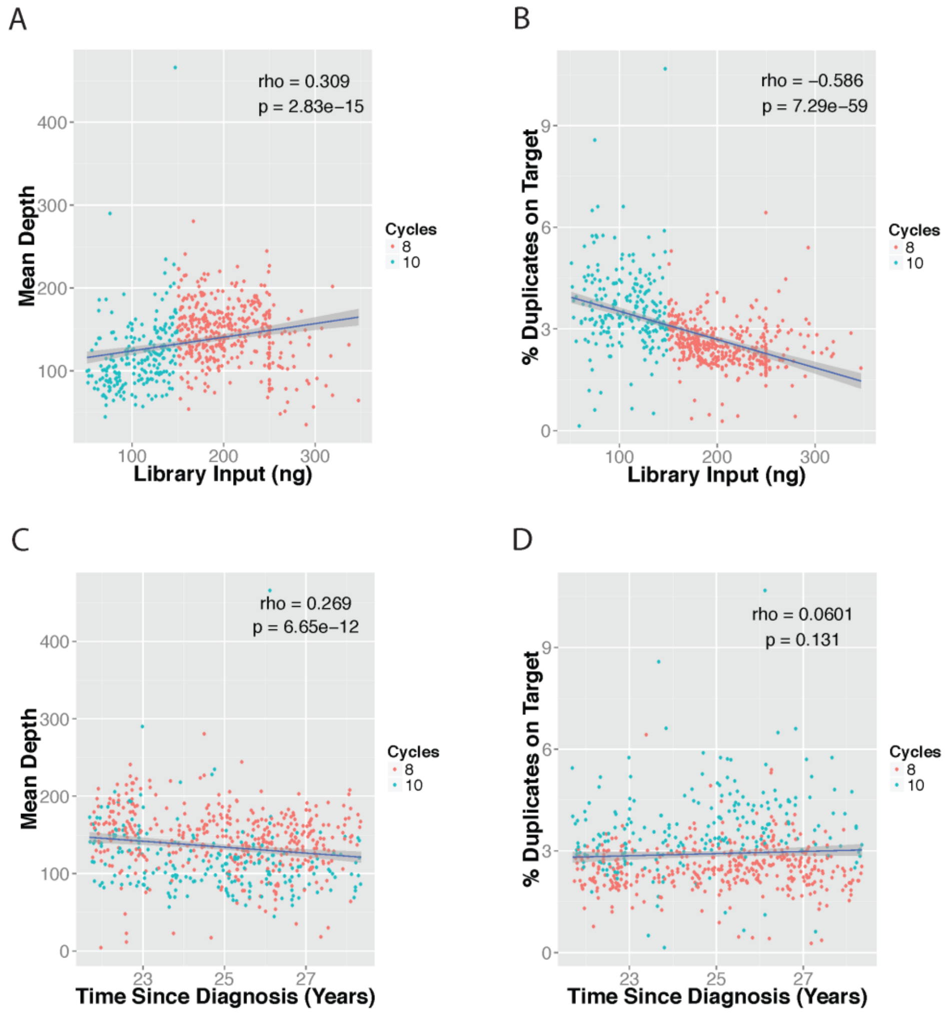

Supplementary Figure 4. Mean Depth and Total Base distributions

The figure below shows the distribution of mean coverage depths (A) and total bases covered (B) across the sample population for TAM, POLAR and MA12 cohorts. Mean coverage was 135.8X (34.7X to 466X) and mean total bases was 336Mb (80.3Mb to 4.94Gb) for TAM cohort.

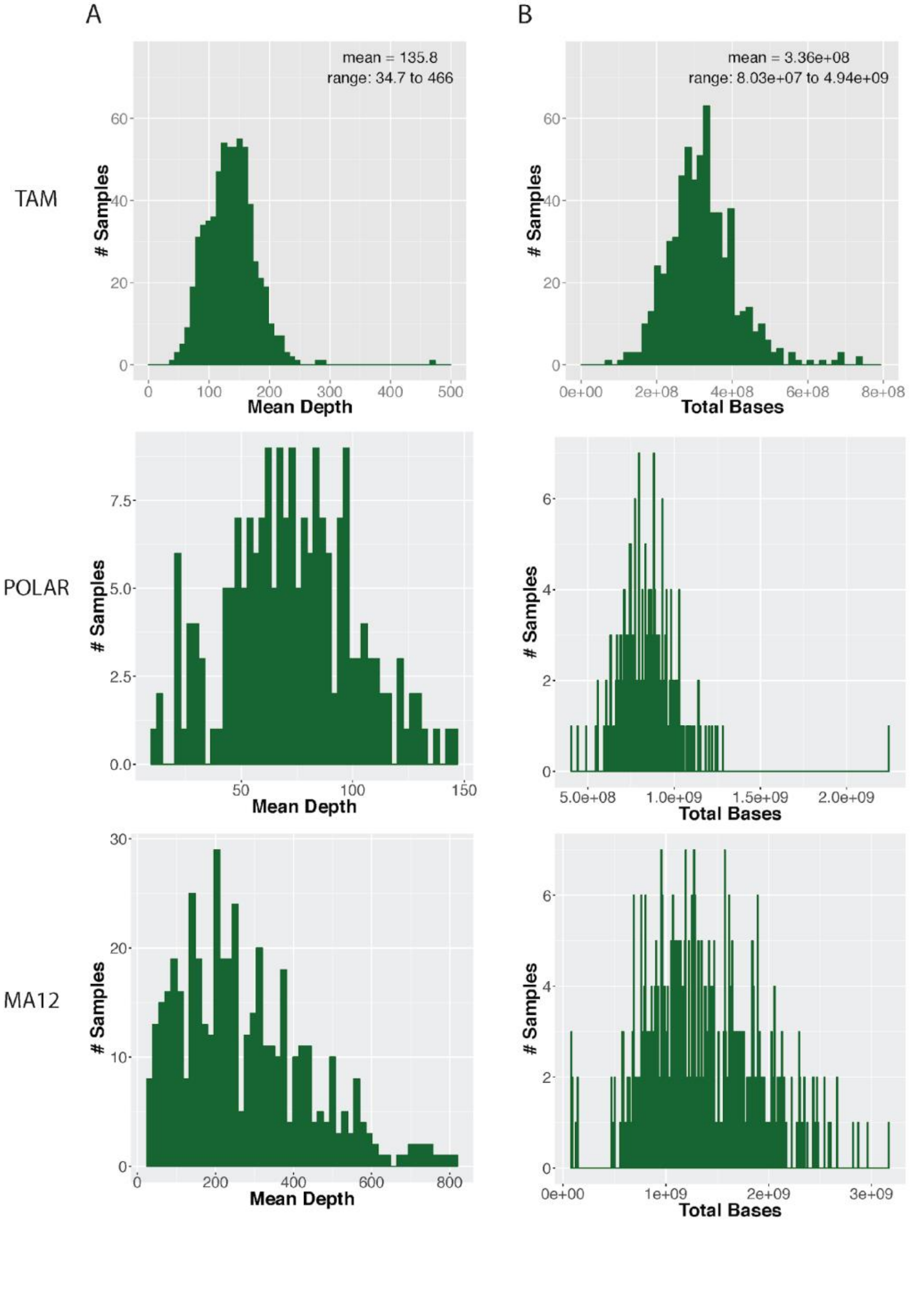

Supplementary Figure 5. Gene-level coverage

To assess gene-by-gene coverage, the median coverage for each gene for each sample was calculated and presented below for the TAM cohort. Genes with a median coverage below 20x are red. The average median coverage at the gene level across all samples was 164x (min 42x for *PIN1* to max 476x for *FRIG1B*).

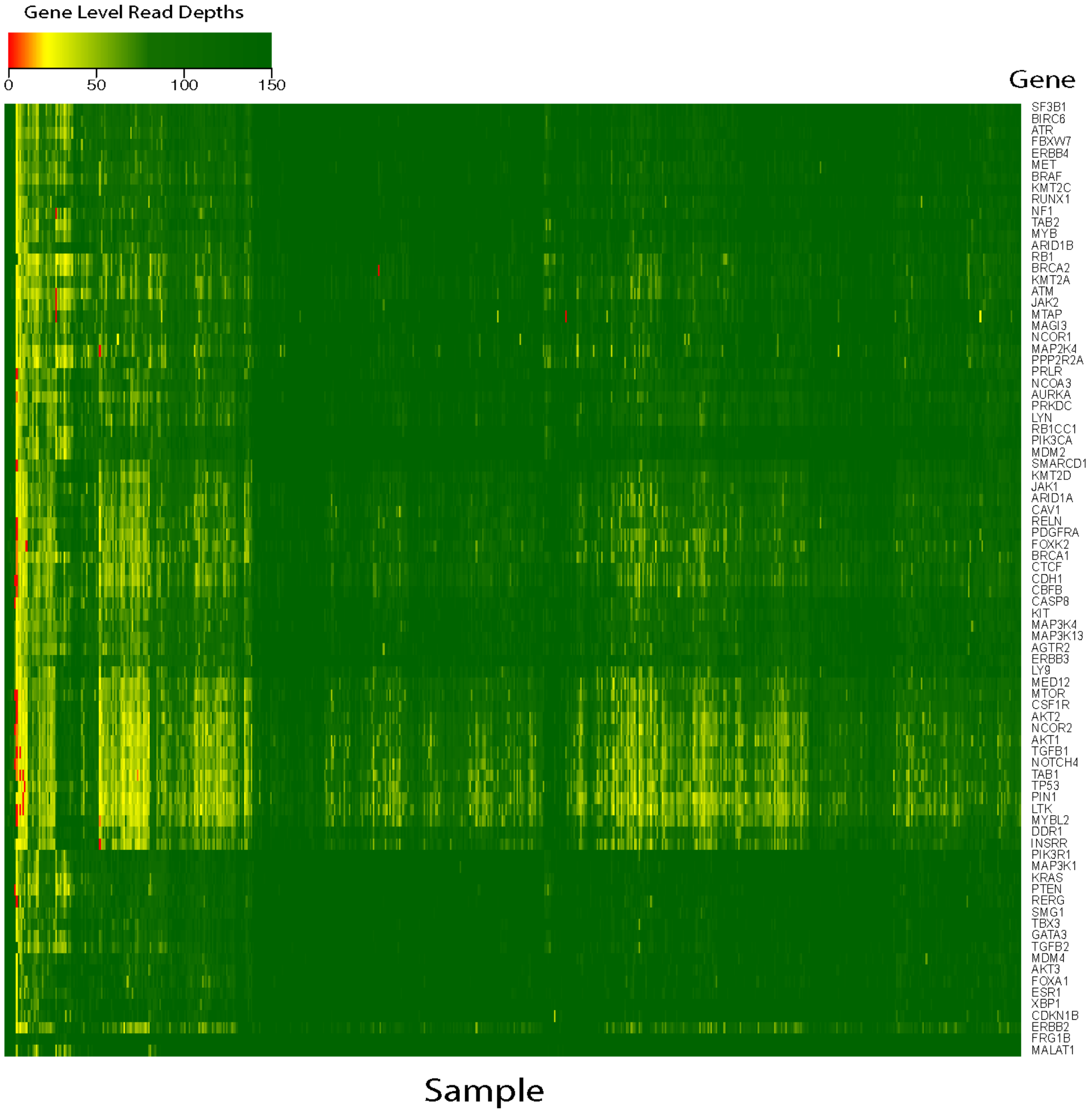

Supplementary Figure 6. Mutation hotspot analysis

Mutation frequency plots illustrate all non-silent mutations for representative Ensembl (70_37) transcripts for several genes of interest: (A) *ESR1* (ENST00000206249), (B) *TP53* (ENST00000269305), (C) *PIK3CA* (ENST00000263967), (D) *MAP3K1* (ENST00000399503), (E) *ARID1B* (ENST00000367148), (F) *NF1* (ENST00000358273). The complete list of mutations for all genes are available in **Supplementary Data 3**.

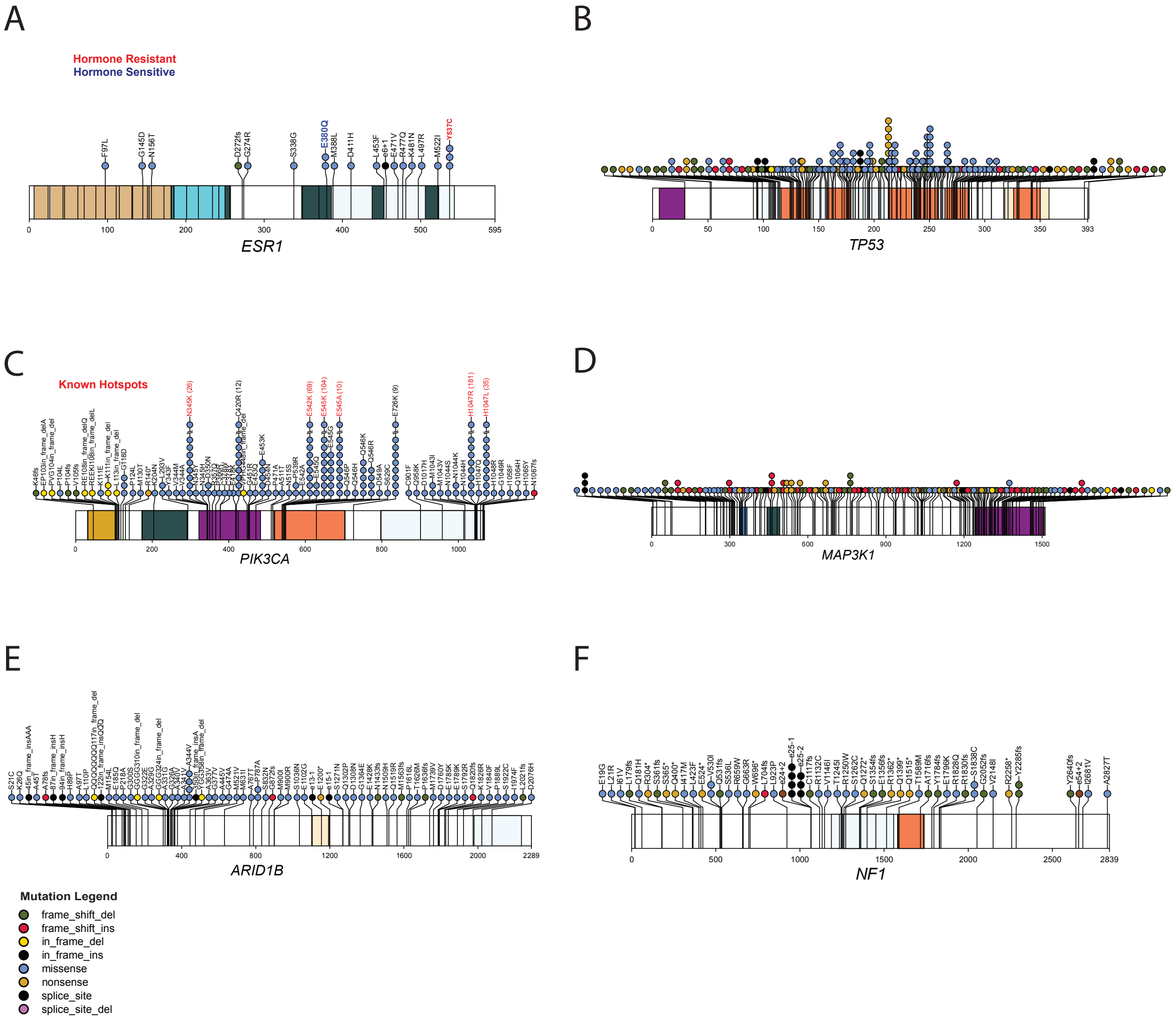

Supplementary Figure 7. Non-silent variants associating with intrinsic subtypes (PAM50) Patient HER2 status by IHC or FISH (+ve vs -ve) and mutation status in NF1 across UBC-TAM+MA12 cohort (left panel) and METABRIC (right panel) (Mut=non-silent mutations) are shown for each PAM50 intrinsic subtype (panel A). NF1 non-silent mutations were found to be enriched in HER2-enriched (HER2-E) HER2 non-amplified cases across both the data sets ($p<0.0001$ and $p=0.003$ respectively). The proportion of patients with non-silent mutations is shown for genes significantly associating with PAM50 in UBC-TAM (panel B) and MA12 (panel C) cohorts. The final table shows the wilcoxon-test p-values for significance of age in GATA3 and ATM Mutated vs wild-type cases (panel D).

A) HER2 IHC/FISH (-ve/+ve) status and mutations in NF1 categorized by intrinsic subtypes

Dataset: UBC-TAM+MA12

PAM50	HER2 -ve		HER2 +ve	
	NF1 WT	NF1 Mut	NF1 WT	NF1 Mut
LuminalA	325	6	19	0
LuminalB	231	5	28	0
Basal	14	1	2	0
HER2-E	43	8	38	0

Dataset: METABRIC

PAM50	HER2 -ve		HER2 +ve	
	NF1 WT	NF1 Mut	NF1 WT	NF1 Mut
LuminalA	622	15	77	2
LuminalB	351	13	121	2
Basal	275	7	46	3
HER2-E	72	8	152	7

B) UBC-TAM

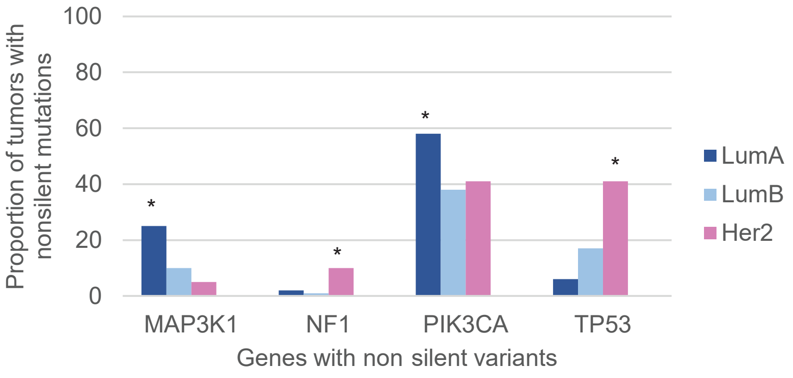

C) MA12

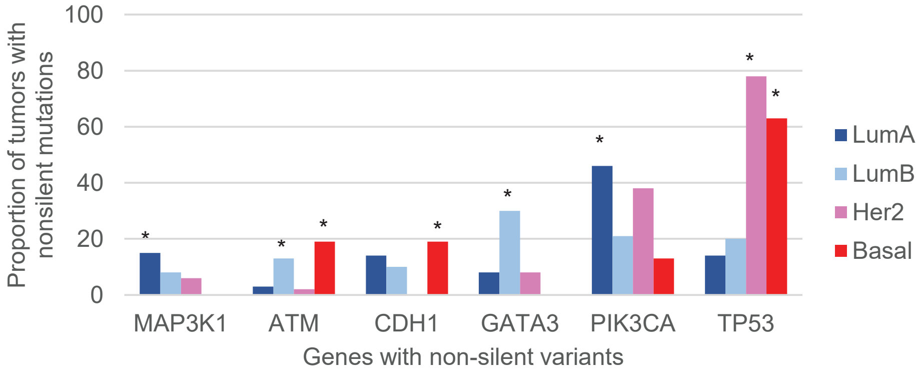

D) Wilcoxon test p-values for significant variance in age (Mut/WT)

Category	#Samples	GATA3	ATM
Luminal	631	0.03	0.31
LumA	361	0.59	0.59
LumB	270	0.01	0.03

Supplementary Figure 8. Survival analysis of activating ERBB2 mutations. Tumors with activating mutations in ERBB2 (red) where compared against ERBB2 wildtype (Black) in two independent ER+ datasets – A) MA12 and B) METABRIC. Also shown is survival curves for tumors with ERBB2 –non-activating (Other) mutations in green.

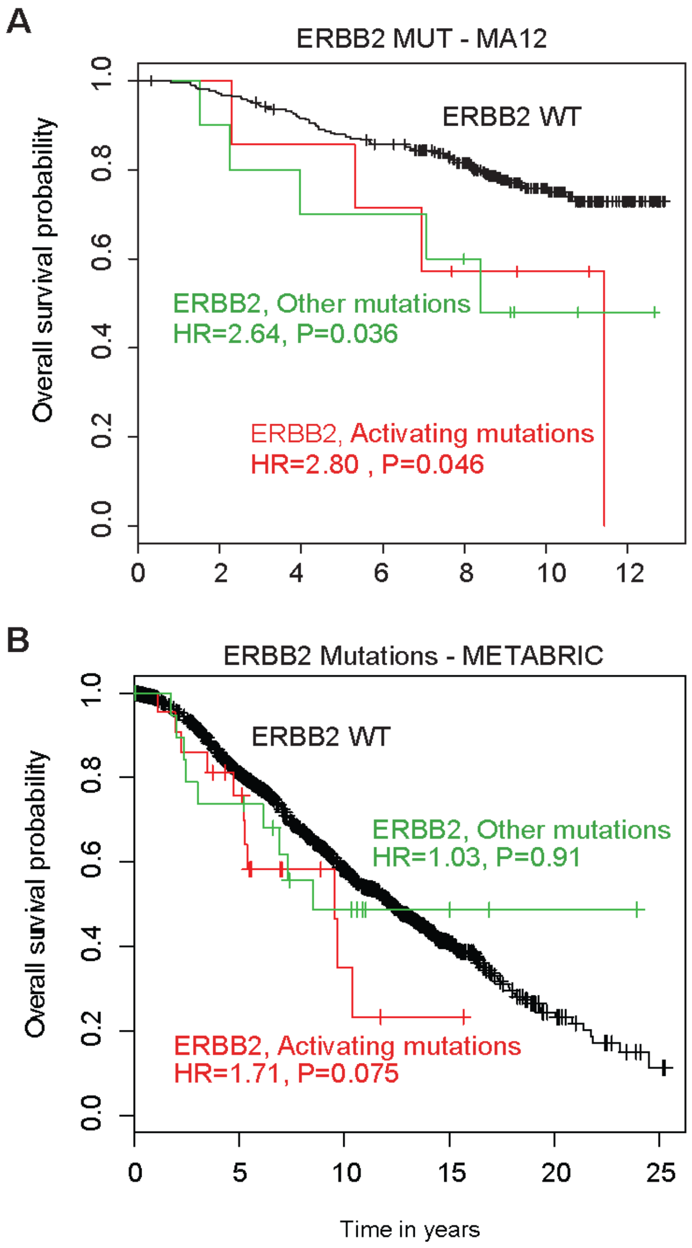

Supplementary Figure 9. Coverage analysis of *CBFB* for TCGA breast exome data

The per-base average coverage, calculated via bedtools (2.17.0) for 410 TCGA breast cancer exomes, are shown across the entire locus (top left) and the 5’ region (exons 1 and 2) of *CBFB* gene (bottom left). Frequency distributions are also shown for the average coverage of the 4 bp exon 2 donor splice site of *CBFB* (top right) and 399 bp of exons 3-6 of *CBFB* (bottom right) for the same TCGA data. The overall average coverage of the exon2 splice site was 4.97 compared to 8.27 for exons 1 and 2 and 97.89 for exons 3 to 6. This lack of coverage of the first two exons of *CBFB* and the corresponding splice sites is the likely reason that this mutation hot spot has gone undiscovered until this study.

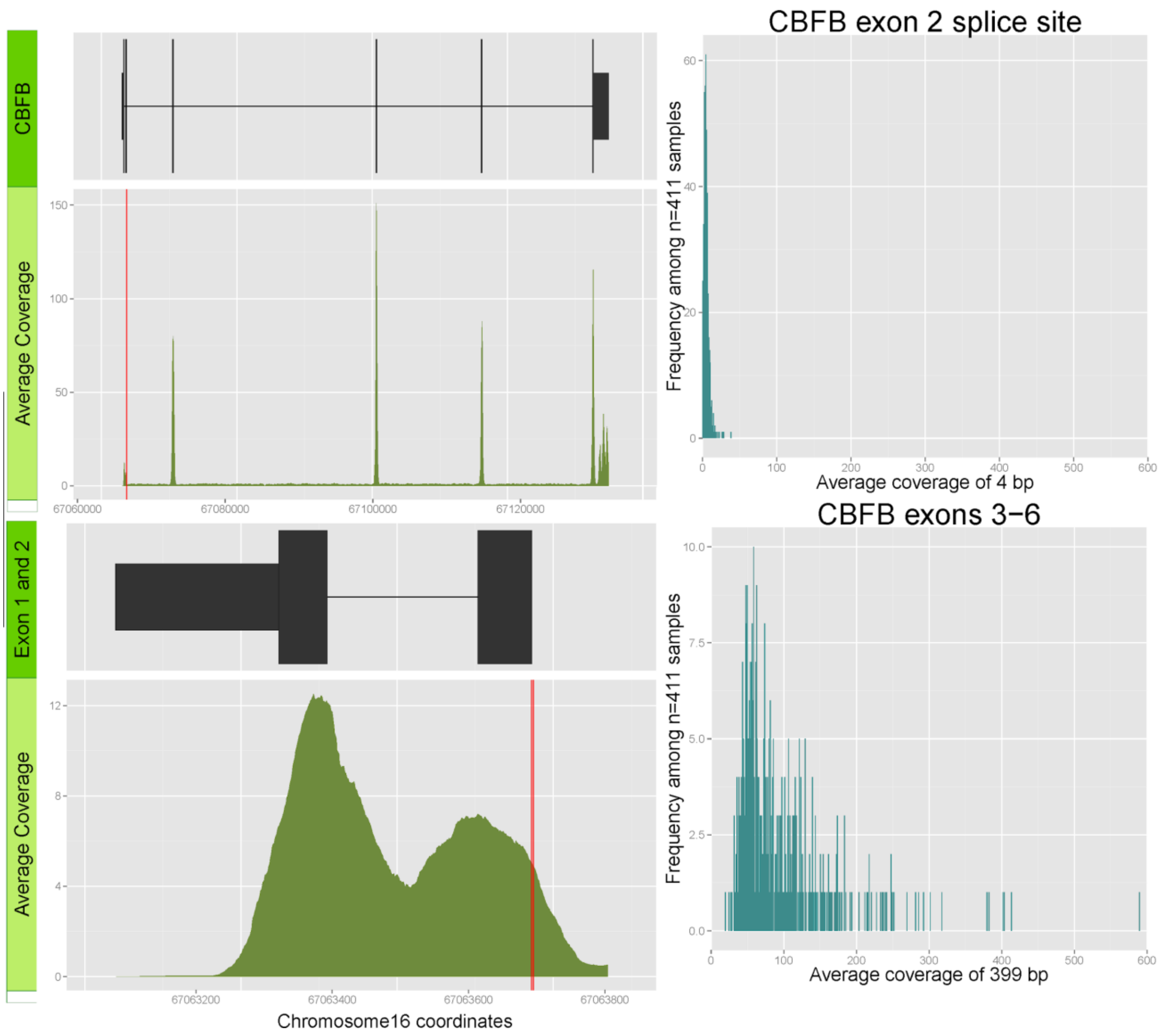

Supplementary Figure 10. GC content of *CBFB* exons relative to all hg19 protein coding exons.

The overall percent of GC content of *CBFB* exons (left) is shown relative to the distribution of percent GC content for all CDS protein coding exons from GRCh37 Ensembl version 74 (right). *CBFB* exon 1 and especially 2 have above average GC content as indicated.

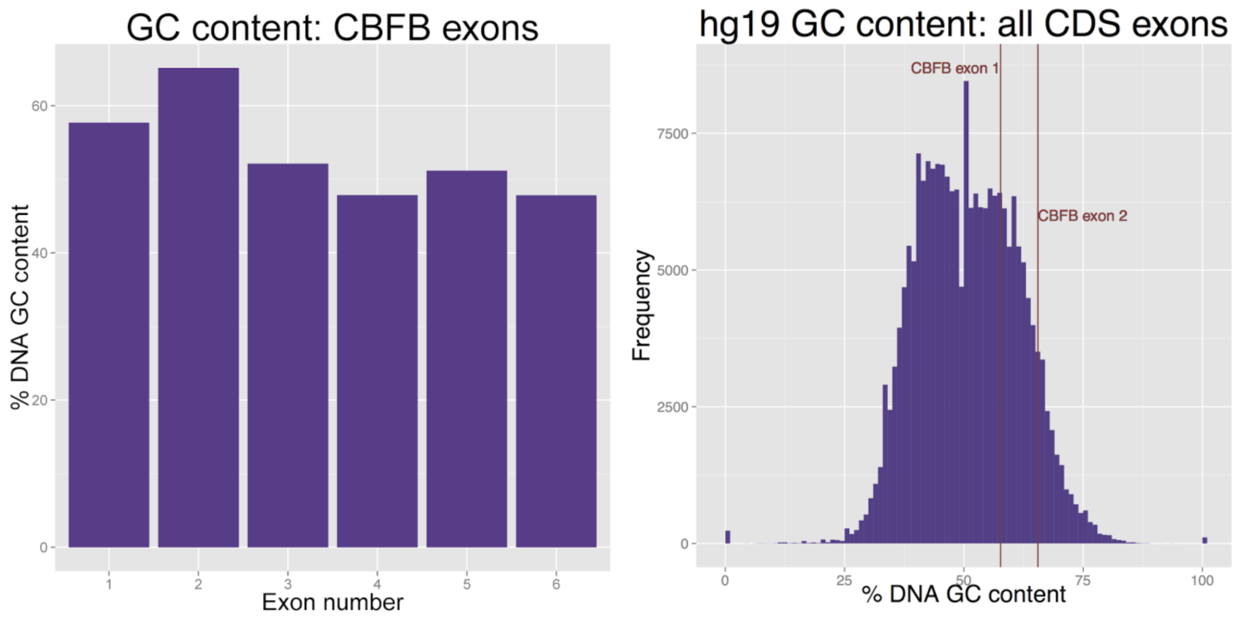

Supplementary Table 1. Gene panel list and associated gene details

EntrezID	Ensembl Gene ID	Gene Biotype	Chr	Gene Start (bp)	Gene End (bp)	HGNC symbol	Synonym
186	ENSG00000180772	protein_coding	X	115301975	115306225	AGTR2	
207	ENSG00000142208	protein_coding	14	105235686	105262088	AKT1	
208	ENSG00000105221	protein_coding	19	40736224	40791443	AKT2	
10000	ENSG00000117020	protein_coding	1	243651535	244014381	AKT3	
8289	ENSG00000117713	protein_coding	1	27022524	27108595	ARID1A	
57492	ENSG00000049618	protein_coding	6	157099063	157531913	ARID1B	
472	ENSG00000149311	protein_coding	11	108093211	108239829	ATM	
545	ENSG00000175054	protein_coding	3	142168077	142297668	ATR	
6790	ENSG00000087586	protein_coding	20	54944445	54967393	AURKA	
57448	ENSG00000115760	protein_coding	2	32582096	32843966	BIRC6	
673	ENSG00000157764	protein_coding	7	140419127	140624564	BRAF	
672	ENSG00000012048	protein_coding	17	41196312	41277500	BRCA1	
675	ENSG00000139618	protein_coding	13	32889611	32973805	BRCA2	
841	ENSG00000064012	protein_coding	2	202098166	202152434	CASP8	
857	ENSG00000105974	protein_coding	7	116164839	116201233	CAV1	
865	ENSG00000067955	protein_coding	16	67063019	67134961	CBFB	
999	ENSG00000039068	protein_coding	16	68771128	68869451	CDH1	
1027	ENSG00000111276	protein_coding	12	12867992	12875305	CDKN1B	
1436	ENSG00000182578	protein_coding	5	149432854	149492935	CSF1R	
10664	ENSG00000102974	protein_coding	16	67596310	67673086	CTCF	
780	ENSG00000204580	protein_coding	6	30844198	30867933	DDR1	
2064	ENSG00000141736	protein_coding	17	37844167	37886679	ERBB2	HER2
2065	ENSG00000065361	protein_coding	12	56473641	56497289	ERBB3	
2066	ENSG00000178568	protein_coding	2	212240446	213403565	ERBB4	
2099	ENSG00000091831	protein_coding	6	151977826	152450754	ESR1	ER
55294	ENSG00000109670	protein_coding	4	153242410	153457253	FBXW7	
3169	ENSG00000129514	protein_coding	14	38059189	38069245	FOXA1	
2296	ENSG00000054598	protein_coding	6	1610681	1614127	FOXC1	
284802	ENSG00000149531	protein_coding	20	29611857	29634010	FRG1B	
8324	ENSG00000155760	protein_coding	2	202899310	202903160	FZD7	
2625	ENSG00000107485	protein_coding	10	8095567	8117161	GATA3	
3645	ENSG00000027644	protein_coding	1	156809855	156828810	INSRR	
3716	ENSG00000162434	protein_coding	1	65298912	65432187	JAK1	
3717	ENSG00000096968	protein_coding	9	4985033	5128183	JAK2	
3815	ENSG00000157404	protein_coding	4	55524085	55606881	KIT	
4297	ENSG00000118058	protein_coding	11	118307205	118397539	KMT2A	MLL
58508	ENSG00000055609	protein_coding	7	151832010	152133090	KMT2C	MLL3
8085	ENSG00000167548	protein_coding	12	49412758	49453557	KMT2D	MLL2

3845	ENSG00000133703	protein_coding	12	25357723	25403870	KRAS
4058	ENSG00000062524	protein_coding	15	41795836	41806085	LTK
4067	ENSG00000254087	protein_coding	8	56792372	56923940	LYN
260425	ENSG00000081026	protein_coding	1	113933371	114228545	MAGI3
378938	ENSG00000251562	lincRNA	11	65265233	65273940	MALAT1
6416	ENSG00000065559	protein_coding	17	11924141	12047147	MAP2K4
4214	ENSG00000095015	protein_coding	5	56111401	56191979	MAP3K1
9175	ENSG00000073803	protein_coding	3	185000729	185206885	MAP3K13
4216	ENSG00000085511	protein_coding	6	161412759	161538417	MAP3K4
4193	ENSG00000135679	protein_coding	12	69201956	69239214	MDM2
4194	ENSG00000198625	protein_coding	1	204485511	204542871	MDM4
9968	ENSG00000184634	protein_coding	X	70338406	70362303	MED12
4233	ENSG00000105976	protein_coding	7	116312444	116438440	MET
4507	ENSG00000099810	protein_coding	9	21802542	21931646	MTAP
2475	ENSG00000198793	protein_coding	1	11166592	11322564	MTOR
4602	ENSG00000118513	protein_coding	6	135502453	135540311	MYB
4605	ENSG00000101057	protein_coding	20	42295754	42345136	MYBL2
8202	ENSG00000124151	protein_coding	20	46130601	46285621	NCOA3
9611	ENSG00000141027	protein_coding	17	15932471	16121499	NCOR1
9612	ENSG00000196498	protein_coding	12	124808961	125052135	NCOR2
4763	ENSG00000196712	protein_coding	17	29421945	29709134	NF1
4855	ENSG00000204301	protein_coding	6	32162620	32191844	NOTCH4
5156	ENSG00000134853	protein_coding	4	55095264	55164414	PDGFRA
5290	ENSG00000121879	protein_coding	3	178865902	178957881	PIK3CA
5295	ENSG00000145675	protein_coding	5	67511548	67597649	PIK3R1
5300	ENSG00000127445	protein_coding	19	9945933	9960358	PIN1
5520	ENSG00000221914	protein_coding	8	26149007	26230196	PPP2R2A
5591	ENSG00000253729	protein_coding	8	48685669	48872743	PRKDC
5618	ENSG00000113494	protein_coding	5	35048861	35230794	PRLR
5728	ENSG00000171862	protein_coding	10	89622870	89731687	PTEN
5925	ENSG00000139687	protein_coding	13	48877887	49056122	RB1
9821	ENSG00000023287	protein_coding	8	53535016	53658403	RB1CC1
5649	ENSG00000189056	protein_coding	7	103112231	103629963	RELN
85004	ENSG00000134533	protein_coding	12	15260717	15501609	RERG
861	ENSG00000159216	protein_coding	21	36160098	37376965	RUNX1
23451	ENSG00000115524	protein_coding	2	198254508	198299815	SF3B1
6602	ENSG00000066117	protein_coding	12	50478755	50494495	SMARCD1
23049	ENSG00000157106	protein_coding	16	18816175	18937776	SMG1
10454	ENSG00000100324	protein_coding	22	39795746	39833065	TAB1
23118	ENSG00000055208	protein_coding	6	149539777	149732749	TAB2
6926	ENSG00000135111	protein_coding	12	115108059	115121969	TBX3

7040	ENSG00000105329	protein_coding	19	41807492	41859816	TGFB1	
7042	ENSG00000092969	protein_coding	1	218519577	218617961	TGFB2	
7157	ENSG00000141510	protein_coding	17	7565097	7590856	TP53	P53
7494	ENSG00000100219	protein_coding	22	29190543	29196585	XBP1	

Supplementary Table 2. Clinical characteristics of cohorts

	**TAM**	**POLAR**	**MA12**
Samples	n = 625	n = 175	n = 328
Age at diagnosis:	67 (40-89+)	57 (20-89+)	45 (30-57)
Tumor size:			
≤ 2 cm	296 (47%)	66 (38%)	140 (43%)
> 2 to 5 cm	300 (48)	99 (57)	162 (49)
> 5 cm	28 (5)	10 (6)	23 (7)
Unknown	1	0	3
Grade:			
Grade 1	22 (4)	18 (10)	98 (30)
Grade 2	265 (42)	78 (45)	148 (45)
Grade 3	307 (49)	79 (45)	59 (18)
Unknown	31 (5)	0	23 (7)
Node status:			
0	156 (25)	91 (52)	58 (18)
1-3	306 (49)	49 (28)	195 (59)
>3	122 (20)	35 (20)	75 (23)
Unknown	41 (6)	0	0
Histology:			
Ductal	561 (90)	132 (75)	328
Lobular	64 (10)	27 (15)	0
Unknown	0	16 (10)	0
PAM50:			
Luminal A	249 (40)	N/A	114 (35)
Luminal B	209 (33)	N/A	61 (19)
Her2	41 (6)	N/A	48 (15)
Basal	2	N/A	15 (5)
Normal-like	6 (1)	N/A	17 (5)
Unknown	118 (19)	N/A	73 (22)

Supplementary Table 3. Novel hotspot analysis

Mutation	mega_mt	komen_mt	wt_mega	wt_komen	fish_p	BH
CBFB e2+1	0	15	1050	1244	0.0001	0.0078
FRG1B E159D	2	20	1048	1239	0.0003	0.0098
PIK3CA E545K	63	104	987	1155	0.0219	0.2624
PIK3CA E542K	39	69	1011	1190	0.0278	0.2629
MAP3K4 A1193in_frame_del	13	30	1037	1229	0.0292	0.2629
NCOR2 511in_frame_insQ	12	27	1038	1232	0.0433	0.2881
CDH1 e10+1	0	5	1050	1254	0.0480	0.2881
PTEN T319fs	2	9	1048	1250	0.0609	0.3373
CDH1 Q23*	3	10	1047	1249	0.0867	0.3515
PIK3CA E545A	3	10	1047	1249	0.0867	0.3515
ARID1B A344V	0	4	1050	1255	0.0882	0.3515
MLL3 P350	0	4	1050	1255	0.0882	0.3515
NF1 e25-1	0	4	1050	1255	0.0882	0.3515
GATA3 H435fs	2	8	1048	1251	0.0939	0.3515
PIK3CA E545G	1	6	1049	1253	0.0976	0.3515
ERBB2 L755S	4	11	1046	1248	0.1124	0.3854
PIK3CA H1047L	23	36	1027	1223	0.1892	0.6193
PIK3CA C420R	6	12	1044	1247	0.2128	0.6663
GATA3 N332fs	1	4	1049	1255	0.2489	0.7467
PIK3CA N345K	17	26	1033	1233	0.2639	0.7599
ERBB2 D769Y	2	4	1048	1255	0.4318	1.0000
AKT1 E17K	30	38	1020	1221	0.4598	1.0000
ERBB2 V777L	3	4	1047	1255	0.5983	1.0000
GATA3 M294K	3	4	1047	1255	0.5983	1.0000
TP53 G266E	3	4	1047	1255	0.5983	1.0000
PIK3CA Q546K	5	5	1045	1254	0.7287	1.0000
TP53 R248Q	5	5	1045	1254	0.7287	1.0000
GATA3 P409fs	12	12	1038	1247	0.7440	1.0000
PIK3CA E726K	10	9	1040	1250	0.8054	1.0000
PIK3CA E453K	6	5	1044	1254	0.8183	1.0000
TP53 I195T	5	4	1045	1255	0.8272	1.0000
PIK3CA G118D	4	3	1046	1256	0.8412	1.0000
TP53 R342*	4	3	1046	1256	0.8412	1.0000
TP53 R248W	8	6	1042	1253	0.8745	1.0000
PIK3CA Q546R	6	4	1044	1255	0.8929	1.0000
TP53 R213*	10	7	1040	1252	0.9120	1.0000
PIK3CA M1043I	4	2	1046	1257	0.9278	1.0000
TP53 E285K	4	2	1046	1257	0.9278	1.0000
TP53 L194R	4	2	1046	1257	0.9278	1.0000
PIK3CA H1047R	176	184	874	1075	0.9296	1.0000
TP53 Y163C	6	3	1044	1256	0.9475	1.0000
TP53 G108fs	5	2	1045	1257	0.9624	1.0000
TP53 G245S	5	2	1045	1257	0.9624	1.0000
TP53 V216M	7	3	1043	1256	0.9709	1.0000
GATA3 R331fs	4	1	1046	1258	0.9807	1.0000
PIK3CA G1049R	4	1	1046	1258	0.9807	1.0000
TP53 C141Y	4	1	1046	1258	0.9807	1.0000

TP53 R306*	5	1	1045	1258	0.9912	1.0000
TP53 R196*	8	2	1042	1257	0.9952	1.0000
TP53 Y220C	12	4	1038	1255	0.9962	1.0000
NCOR2 S1840fs	7	1	1043	1258	0.9982	1.0000
TP53 H179R	7	1	1043	1258	0.9982	1.0000
TP53 R273H	7	1	1043	1258	0.9982	1.0000
TP53 R273C	8	1	1042	1258	0.9992	1.0000
TP53 R175H	18	4	1032	1255	0.9999	1.0000
FRG1B 57in_frame_insL	5	0	1045	1259	1.0000	1.0000
FRG1B H8Y	7	0	1043	1259	1.0000	1.0000
FRG1B L153P	5	0	1045	1259	1.0000	1.0000
GATA3 e4-2	19	0	1031	1259	1.0000	1.0000
MAP2K4 S184L	4	0	1046	1259	1.0000	1.0000
NCOA3 Q1257in_frame_del	4	0	1046	1259	1.0000	1.0000
NCOA3 Q1258in_frame_del	15	0	1035	1259	1.0000	1.0000
NCOR2 1846in_frame_insSSG	6	0	1044	1259	1.0000	1.0000
SF3B1 K700E	16	0	1034	1259	1.0000	1.0000
TP53 C176F	5	0	1045	1259	1.0000	1.0000
TP53 G245D	4	0	1046	1259	1.0000	1.0000
TP53 H193R	10	0	1040	1259	1.0000	1.0000

Supplementary Table 4. Univariate and multivariate analysis of TAM candidates in hormone-therapy-treated post-menopausal METABRIC subjects

Anaysis	Gene	VariationT	HR	P	lower	upper
1UVA	ARID1B	non-silent	0.823	0.68	0.332	2.041
MVA	ARID1B	non-silent	0.729	0.49	0.294	1.807
1UVA	ERBB3	non-silent	0.8846	0.787	0.360	2.156
MVA	ERBB3	non-silent	0.8397	0.701	0.344	2.053
1UVA	MAP3K1	non-silent	0.751	0.239	0.466	1.211
MVA	MAP3K1	non-silent	0.771	0.294	0.475	1.253
1UVA	NF1	FS/NS	2.44	0.049	1.000	5.940
MVA	NF1	FS/NS	3.38	0.008	1.380	8.317
1UVA	PIK3CA	non-silent	1.03968	0.7954	0.775	1.395
MVA	PIK3CA	non-silent	1.02699	0.86327	0.758	1.391
1UVA	TP53	non-silent	2.1968	1.62E-07	1.624	2.972
MVA	TP53	non-silent	2.0829	6.63E-06	1.514	2.866
clinical	Tumor Grade	Clinical	1.5277	0.005697	1.131	2.063
clinical	Node Positivity	Clinical	3.032	9.14E-09	2.077	4.426
clinical	Tumor Size >5cm	Clinical	4.076	1.20E-07	2.423	6.858

Supplementary Table 5. Analysis of effect of considering CNV status of selected amplification status on mutation-prognosis associations

Dataset	Analysis	Analysis_type	Gene	Variation_type	HR	P	lower	upper
METABRIC_forTAM	MVA:AMP-ERBB2,FGFR1,CCND1,MYC	BCSS	ARID1B	non-silent	0.727	0.485087	0.297	1.779
METABRIC_forTAM	MVA:AMP-ERBB2,FGFR1,CCND1,MYC	BCSS	ERBB3	non-silent	0.839	0.649144	0.394	1.787
METABRIC_forTAM	MVA:AMP-ERBB2,FGFR1,CCND1,MYC	BCSS	MAP3K1	non-silent	0.6124	0.04324	0.381	0.985
METABRIC_forTAM	MVA:AMP-ERBB2,FGFR1,CCND1,MYC	BCSS	NF1	Truncating	2.1325	0.069195	0.942	4.826
METABRIC_forTAM	MVA:AMP-ERBB2,FGFR1,CCND1,MYC	BCSS	PIK3CA	non-silent	1.07086	0.609028	0.824	1.392
METABRIC_forTAM	MVA:AMP-ERBB2,FGFR1,CCND1,MYC	BCSS	TP53	non-silent	2.0428	6.71E-07	1.541	2.708
METABRIC_forMA12	MVA:AMP-ERBB2,FGFR1,CCND1,MYC	OS	ERBB2	nonsilent	1.38267	0.15531	0.884	2.162
METABRIC_forMA12	MVA:AMP-ERBB2,FGFR1,CCND1,MYC	OS	ERBB4	nonsilent	0.74375	0.4392	0.351	1.575
METABRIC_forMA12	MVA:AMP-ERBB2,FGFR1,CCND1,MYC	OS	JAK1	MS	1.418429	0.19711	0.834	2.413
METABRIC_forMA12	MVA:AMP-ERBB2,FGFR1,CCND1,MYC	OS	PIK3R1	nonsilent	1.83177	0.00955	1.159	2.895
METABRIC_forMA12	MVA:AMP-ERBB2,FGFR1,CCND1,MYC	OS	PIK3R1	truncating	2.24241	0.01161	1.198	4.198

METABRIC_forMA12	MVA:AMP-ERBB2,FGFR1,CCND1,MYC	OS	RB1	nonsilent	1.19135	0.4926	0.723	1.964
