# Supplementary material for: The prognostic effects of somatic mutations in ER-positive breast cancer
Source: Nat Commun. 2018 Sep 4;9:3476. doi: 10.1038/s41467-018-05914-x (PMC6123466; doi:10.1038/s41467-018-05914-x)
Supplement: Supplementary file 3 — Description of Additional Supplementary Files [file 41467_2018_5914_MOESM3_ESM.pdf]

Description of Additional Supplementary Files

File Name: Supplementary Data 1
Description: Multi-study MAF file summarizing mutation rates across six studies used for gene selection and hotspot analysis

File Name: Supplementary Data 2
Description: Sequence coverage data

File Name: Supplementary Data 3
Description: Final MAF file of all mutations for 83 targeted gene study

File Name: Supplementary Data 4
Description: Mutual exclusivity and mutual occurrence data

File Name: Supplementary Data 5
Description: Subtype associations with mutation status in UBC-TAM and MA12

File Name: Supplementary Data 6
Description: Recurrence and breast cancer specific univariate survival for genes based on mutation type

File Name: Supplementary Data 7
Description: Functional analysis of rare mutations

File Name: Supplementary Data 8
Description: Targeted genes and probes

File Name: Supplementary Data 9
Description: BRCA and ATM pathogenicity analysis
